# Elucidating and Optimizing I Occupation in Lithium Argyrodite Solid Electrolytes for Advanced All‐Solid‐State Li Metal Batteries

**DOI:** 10.1002/EXP.20240050

**Published:** 2025-08-25

**Authors:** Zhikai Huang, Wenrui Sun, Shuaiyu He, Huan Hu, Xue Li, Ke Huang, Zhihao Yan, Gencai Guo, Yaojie Lei, Liwen Yang, Jianyu Huang, Gang Wang, Yaru Liang, Guobao Xu, Xingqiao Wu

**Affiliations:** ^1^ Hunan Provincial Key Laboratory of Thin Film Materials and Devices School of Material Sciences and Engineering Xiangtan University Xiangtan China; ^2^ School of Physics and Optoelectronics Xiangtan University Xiangtan China; ^3^ Centre for Clean Energy Technology University of Technology Sydney Sydney New South Wales Australia; ^4^ Institute for Carbon Neutralization Technology College of Chemistry and Materials Engineering Wenzhou University Wenzhou China

**Keywords:** iodine‐rich Li_6−_ *_x_* PS_5−_ *_x_* ClI*_x_*, high ionic conductivity, all‐solid‐state lithium batteries

## Abstract

Although iodine (I) doped Li_6_PS_5_Cl argyrodite sulfide electrolytes have attracted significant attention, a comprehensive understanding of how I^−^ occupancy influences ionic conductivity is still lacking. Herein, through ab initio molecular dynamics theoretical calculations, it was revealed that the incorporation of excess halogen at the sulfur site (4d) significantly accelerates the inter‐cage jumps of Li^+^ with a low migration energy barrier of 0.28 eV, enhancing the ionic diffusion kinetics. Subsequently, iodine‐rich Li_6−_ *_x_* PS_5−_ *_x_* ClI_x_ (0 ≤ *x* ≤ 0.2) electrolytes are successfully synthesized and deliver high ionic conductivity. Moreover, a stable Li/Li_6−_ *_x_* PS_5−_ *_x_* ClI*_x_* interface is achieved to inhibit side reactions and lithium dendrite growth. Therefore, Li symmetric cells with the optimized electrolyte present splendid cyclic stability (7000 h at 0.1 mAh cm^−2^ and 1500 h at 0.5 mAh cm^−2^). The constructed full cells with optimized electrolytes exhibit excellent electrochemical properties at a broad temperature range and with different active materials. This work deepens the understanding of the relationship between ion transport and structure in lithium argyrodite sulfide electrolytes.

## Graphical abstract

Through ab initio molecular dynamics theoretical calculations, we reveal that incorporation of excess halogen at sulfur site (4d) accelerates inter‐cage jumps of Li^+^ with a low migration energy barrier. Subsequently, iodine‐rich Li_6−_ *_x_* PS_5−_ *_x_* ClI*_x_* (0 ≤ *x* ≤ 0.2) electrolytes with high ionic conductivity are successfully synthesized. Therefore, all‐solid‐state batteries with LPSC‐0.1LiI electrolyte deliver excellent electrochemical properties.

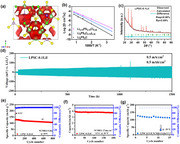

## 1 Introduction

Compared with rechargeable lithium‐ion batteries based on liquid electrolytes [1, 2], all‐solid‐state batteries (ASSBs) have been regarded as ideal next‐generation energy storage devices due to their desirable safety [3, 4, 5], high energy density, and wide temperature operating stability [6, 7, 8]. Recently, sulfide‐based solid‐state electrolytes (SSEs), especially the Li_6_PS_5_X (X = Cl, Br, and I) argyrodites sulfide SSEs with the cubic structure (F‐43m space group), are particularly attractive because of their high ionic conductivity (>10^−3^ S cm^−1^), adaptable processability, favorable mechanical properties, and earth‐abundant precursors [9, 10]. Unfortunately, achieving high ionic conductivity of argyrodite sulfide SSEs remains a challenge when compared to commercial liquid electrolytes (≈10^−2^ S cm^−1^). To conquer the barrier, researchers have employed element doping strategy in this structure type to enhance the ionic conductivity, including the introduced Li‐vacancies on the Li‐site through aliovalent substitution (Al^3+^[11] and Ca^2+^[12]), P‐site disorganization via element substitution (Si [11], Ge [13], Sb [14], and Sn [15]), lattice softening using chalcogenides substitution (Se [16] and Te [17]), large‐scale 4d site reorganization induced through excess halogen substitution (Cl [18], Br [19], and I [20]), and co‐substitution strategy (In/O [21], Sn/I [22] co‐doping etc.).

Recently, due to the significant influence of positional exchange between halogens at the 4a (0,0,0) site and sulfur at the 4d (0.25, 0.25, 0.75) site on ionic hopping rate, the introducing different degrees of X^−^/S^2−^ site disorder in the Li_6_PS_5_Cl (LPSC) has been extensively researched as an effective method to improve the properties of SSEs, in which the X^−^ anion and S^2−^ occupy the face‐centered cubic (fcc) lattice, with the PS_4_^3−^ tetrahedra filling the octahedral positions [23, 24]. For instance, based on the molecular dynamics simulation, Wagemaker et al. predicted that 75% 4d Cl site disorder would yield the highest ionic conductivity [25]. On the other hand, the various halogen‐rich lithium argyrodites sulfide SSEs such as Li_5.5_PS_4.5_Cl_1.5_ [26], LPSCl_0.3_F_0.7_ [27], Li_5.5_PS_4.5_Cl*_x_* Br_1.5−_ *_x_* (0 ≤ *x* ≤ 1.5) [19] have been successfully synthesized and exhibited improved performance. It is worth noting that the ionic radius of I^−^ is larger than those of Br, Cl, leading to a hard exchange of S/I at the 4d position [10, 20]. Interestingly, Yan et al. recently demonstrated that increased I^−^ content could promote the ionic conductivity of the LPSC electrolyte, suggesting the possibility of ionic exchange [28]. However, a thorough understanding of how the occupancy of I^−^ affects ionic conductivity is lacking.

In this work, the significance of S/I substitution in lithium argyrodites SSEs for improving ionic conductivity and interfacial stability was explored through collaborative calculation and experimental methods. Utilizing ab initio calculations of molecular dynamics (AIMD) explore the impact of different substitutions of I on lithium ion transport and pathways in SSEs. The findings revealed that disclosing I at the 4d site leads to the formation of interconnected lithium structural domains, thereby converting previously isolated inter‐cage hopping processes into expansive networks facilitating lithium ion diffusion. Guided by the simulation results, the I‐substituted LPSC electrolyte with 10% level at the 4d position (LPSC‐0.1LiI) was prepared by optimizing the annealing process and delivered a high ionic conductivity (4.2 mS cm^−1^ at room temperature). The in situ formation of a stable Li/LPSC‐0.1LiI interface was investigated using scanning electron microscopy (SEM) and X‐ray photoelectron spectroscopy (XPS), which can effectively promote during electrochemical cycling and inhibit lithium dendrite growth. Thus, the Li symmetric cell with LPSC‐0.1LiI exhibited an ultra‐long cycle life of over 1500 h at the current density of 0.5 mA cm^−2^ and a high capacity of 0.5 mAh cm^−2^, the ASSBs with LPSC‐0.1LiI achieved stable long cycling over a wide temperature range.

## 2 Results and Discussion

### 2.1 Theory‐Oriented Design

**FIGURE 1:**
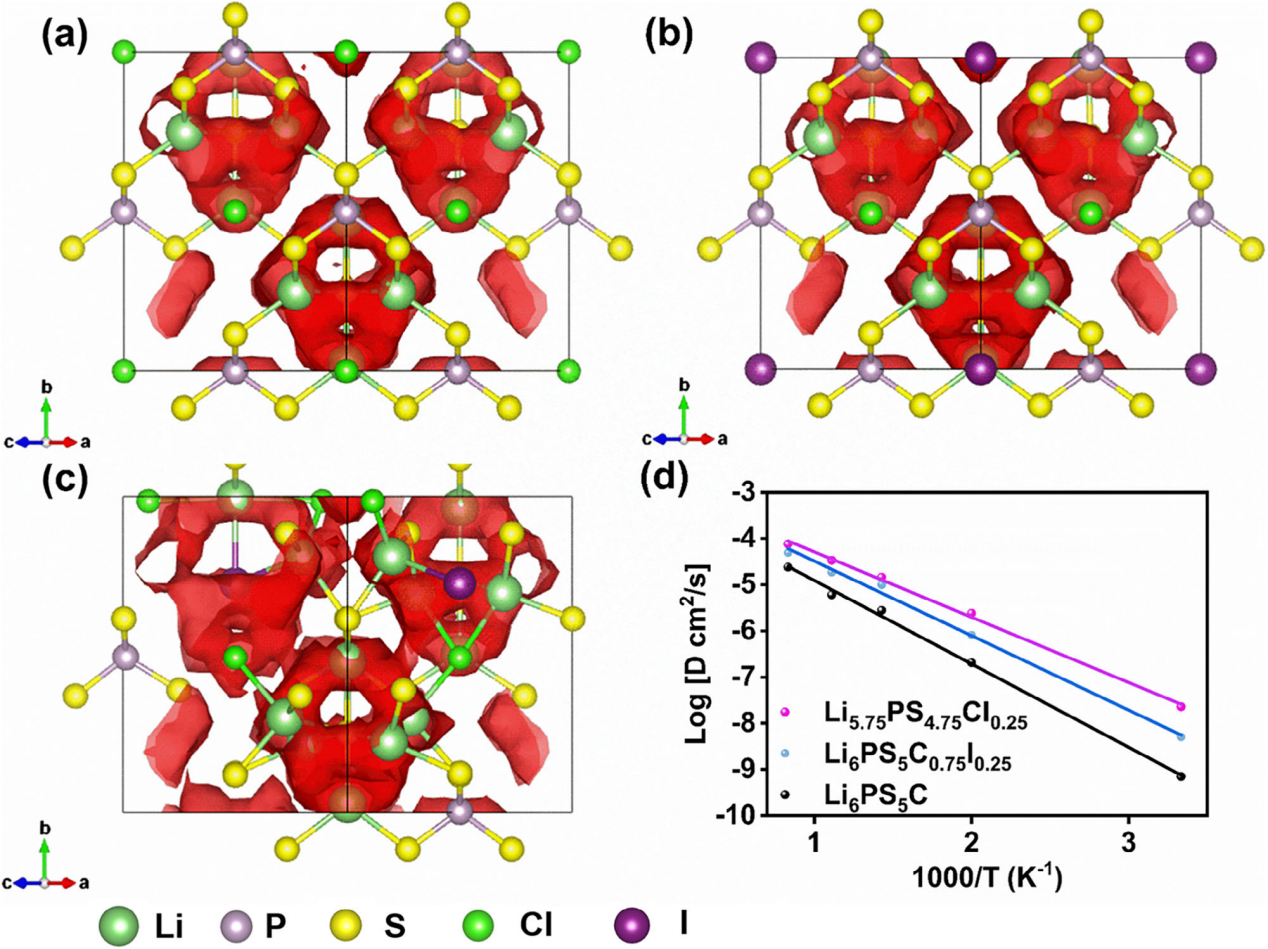
Li diffusion analyzed using AIMD simulations at 900 K. Isosurfaces of the Li‐ion probability densities of (a) LPSC, (b) LPSC_0.75_I_0.25_, and (c) LPS_4.75_CI_0.25_. (d) Calculated Li diffusivity at various temperatures of LPSC, LPSC_0.75_I_0.25_, and LPS_4.75_CI_0.25_.

In order to investigate the influence of mixed anionic lattice on the structural patterns and lithium ion conduction, the AIMD theoretical calculations were carried out to explore two potential substitution sites for the I^−^ (heterovalent I^−^ substituted S^2−^ and isovalent substitution of I with Cl). As shown in Figure 1, the AIMD calculations uncover a confined “cage‐like” lithium diffusion mechanism in pristine LPSC, characterized by constrained Li^+^ movement within Li_6_S octahedral cages. This confinement results in predominantly localized Li^+^ displacements within the LPSC crystal structure, thereby explaining the original poor macroscopic ionic conduction of LPSC (Figure 1a). Similar to LPSC, Li_6_PS_5_Cl_0.75_I_0.25_ also exhibits limited short‐range “cage‐like” lithium diffusion (Figure 1b), but this diffusion is found to be stronger than that in the LPSC lattice from the simulation results. In contrast, the Li_5.75_PS_4.75_ClI_0.25_ structure, where I^−^ substituted S^2−^, presents interconnected Li domains forming diffusion channels with inter‐cage hopping between pairs of 48 h sites due to the activation of the inter‐cage pathway as illustrated in Figure 1c. Therefore, the Li_5.75_PS_4.75_ClI_0.25_ will deliver a significantly improved ionic conductivity, making it a promising candidate for an outstanding electrolyte. In addition, the AIMD simulations were also conducted to analyze the diffusion characteristics of electrolytes over a temperature range of 300–1200 K. The activation energy (*E*_a_) for ion transport in electrolytes is determined by measuring temperature‐dependent ionic conductivity (*σ*) as depicted in Figure 1d. The activation energy of LPSC (0.36 eV) was calculated and aligns with the value in previous literature [29]. In contrast, the activation energies of Li_6_PS_5_Cl_0.75_I_0.25_ and Li_5.75_PS_4.75_ClI_0.25_ were reduced to 0.32 and 0.28 eV, respectively, suggesting that the substitution of I into the lattice structure facilitates Li^+^ migration, thereby enhancing diffusion rates and ionic conductivity. Particularly, the structural modification via I^−^ substituted S^2−^ plays a crucial role in heightening ionic diffusion kinetics.

### 2.2 Synthesis and Structural Characterization of SSEs

**FIGURE 2:**
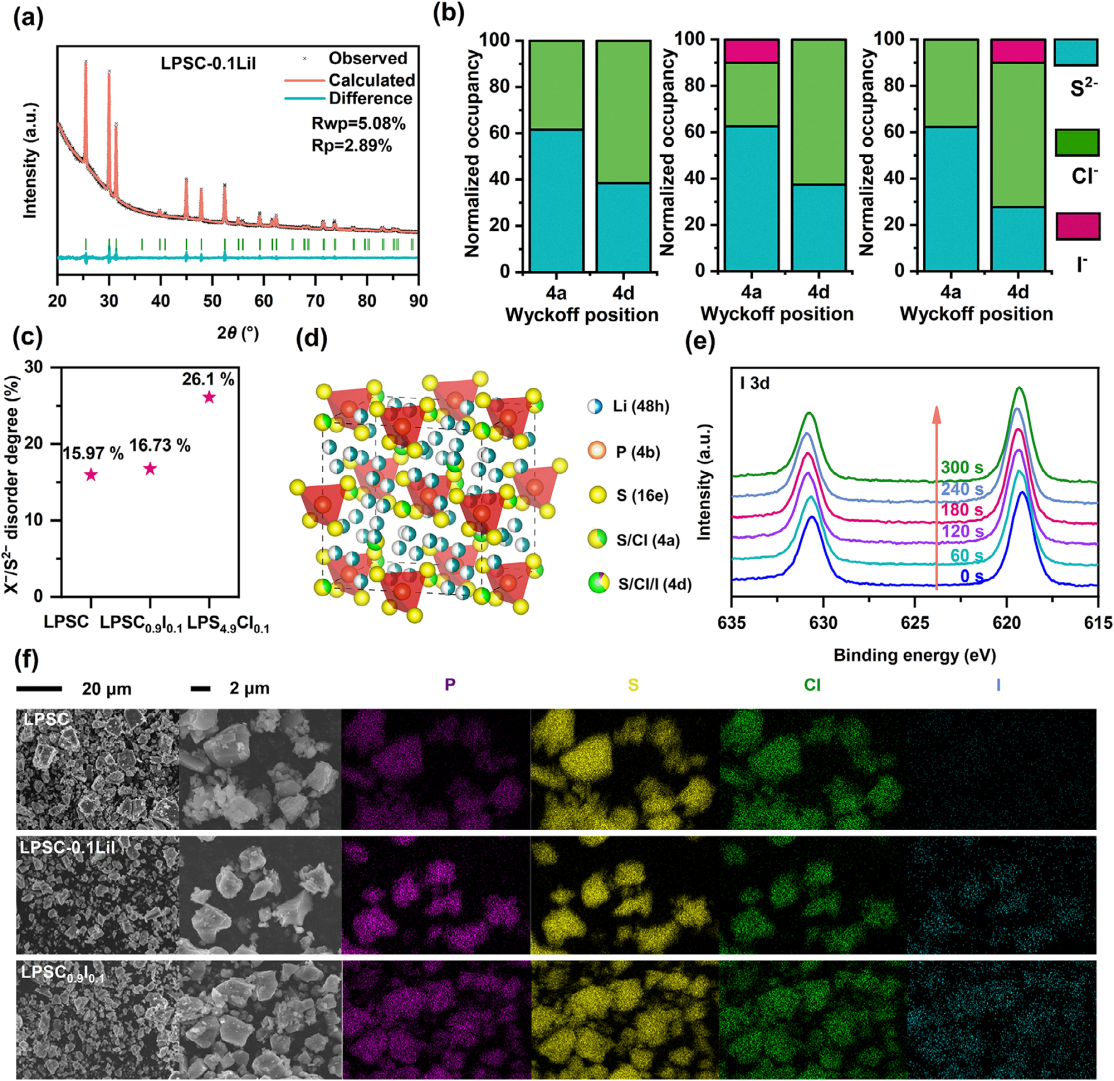
(a) XRD pattern and the corresponding Rietveld refinement result of LPSC‐0.1LiI. (b) Normalized occupancies of the different crystallographic sites of LPSC, LPSC_0.9_I_0.1_, and LPSC‐0.1LiI. (c) The X^−^/S^2−^ degree of disorder in LPSC, LPSC_0.9_I_0.1_, and LPSC‐0.1LiI. (d) Schematic crystal structure of LPSC‐0.1LiI. (e) I 3d with XPS depth profiling measurements of LPSC‐0.1LiI. (f) The SEM images and corresponding EDS mappings of LPSC, LPSC_0.9_I_0.1_, and LPSC‐0.1LiI.

Guided by theoretical calculations, a series of argyrodites Li_6−_ *_x_* PS_5−_ *_x_* ClI*_x_* (*x* = 0, 0.05, 0.1, 0.15, and 0.2) were synthesized to investigate the influence of mixed‐anion lattice on the argyrodite structure and ionic conduction, and characterized via XRD as depicted in Figure S1a. All the as‐prepared electrolytes are dominated by argyrodite structure (space group F‐43m), confirming the successful synthesis of the target lithium argyrodite phase. In comparison, the overall lattice structure of samples almost remains unchanged after increasing the I concentration, but the peak position is systematically shifted toward lower diffraction angles (2*θ*) due to the larger atomic radius of I than that of S (Figure S1b), suggesting a slightly increased lattice constant. In stark contrast to substitutions of Br or Cl, elevating the value of *x* beyond 0.15 results in the formation of two phases [19, 26], which could be ascribed to the proclivity of surpassing the solid solution's solubility threshold because of the large ionic radius of I. According to the stoichiometric ratio, it will form the LPSI phase with Li_2_S and P_2_S_5_ rather than continue to generate solid‐solution behavior using excess LiI. The Raman spectrum was employed to elucidate the structure of the synthesized SSEs. Both pristine LPSC and Li_6−_ *_x_* PS_5−_ *_x_* ClI*_x_* SSEs (Figure S2) exhibit Raman bands at around 429 cm^−1^ assigned to the stretching vibration mode of PS_4_^3−^ units. Additionally, the peaks at wavenumbers 196, 267, 574, and 603 cm^−1^ correspond to the deformation vibration and asymmetric stretching vibration of PS_4_^3−^, indicating identical argyrodite structures [30, 31]. The crystal structure information of the Li_5.9_PS_4.9_ClI_0.1_ (LPSC‐0.1LiI) and LPSC electrolytes, as determined by Rietveld refinement analysis [32], is presented in Figure 2a and Figure S3, and the detailed refinement data are provided in Tables S1 and S2, where the iodine occupies at the 4d position, replacing part of the S and generating Li vacancies.

Meanwhile, Li_6_PS_5_Cl_0.9_I_0.1_ (LPSC_0.9_I_0.1_) electrolyte was synthesized, where iodine substitutes for chlorine while maintaining the identical iodine proportion. The XRD and Raman spectroscopic results, presented in Figure S4, confirm that the electrolyte still maintains the argyrodite‐type crystal structure. Notably, the XRD refinement analysis [33], as illustrated in Figure S5 and Table S3, reveals that iodine predominantly occupies the 4a site within the crystal lattice. Figure 2b,c illustrates the normalized occupancy of S^2−^, Cl^−^, and I^−^ at varying positions within the three previously mentioned electrolytes [34]. In the LPSC system, there is a mutual occupancy of both sulfide and chloride ions at the Wyckoff 4a and 4d sites. Specifically, 15.97% of the Wyckoff 4d sites are occupied by chlorine ions, where the fraction is defined as the disorder degree of X^−^/S^2−^. The substitution of LiI for Li_2_S resulted in an increase in halogen disorder at the 4d site to 26.1%, indicating heightened disorder in LPSC‐0.1LiI. The increased disorder could potentially result in a flattening of the energy landscape and a reduced activation energy barrier [35]. Conversely, the escalation in disorder did not manifest upon substituting LiI for lithium chloride (LiCl).

X‐ray photoelectron spectroscopy (XPS) was utilized to evaluate the chemical states of various elements in structural units of electrolytes. As shown in Figure S6, the S 2p spectrum presents two characteristic peaks at 161.7 and 163.4 eV corresponding to Li‐S‐P and P‐S‐P, respectively, in both LPSC and LPSC‐0.1LiI. The PS_4_^3−^ groups are also evident in the spectrum of P 2p, exhibiting binding energies of 131.2 and 133.1 eV [36]. Moreover, the characteristic peaks of Cl 2p and Li 1s are hardly consistent in both pristine LPSC and LPSC‐0.1LiI, as depicted in Figure S6. These results imply that I doping primarily affects the crystallographic structure and phase behavior of the electrolyte, without substantially modifying the core chemical environments of the constituent elements. To confirm the homogeneous iodine doping in LPSC‐0.1LiI, the XPS depth profiling measurement was performed using an Ar500+ cluster ion source for 60 s at regular intervals as shown in Figure 2e, in which a distinct peak corresponding to the characteristic I_3d_ peaks is evident after iodine doping and the intensity remains consistent even as the etching depth increases, indicating that the iodine doping is uniform throughout the depth of the sample.

The surface morphology and elemental distribution of the SSEs were examined using SEM and energy dispersive spectroscopy (EDS). As illustrated in Figure 2f, the SEM images reveal that the incorporation of iodine did not significantly alter the morphology of the electrolytes. Each particle with a micrometer size range appears to be an aggregation of smaller particles. Furthermore, the EDS mapping results confirm the uniform distribution of elements such as phosphorus (P), sulfur (S), chlorine (Cl), and iodine (I) throughout the LPSC‐0.1LiI electrolyte, suggesting that iodine is integrated into the electrolyte without forming aggregates or secondary phases.

### 2.3 Electrochemical Performances of SSEs

**FIGURE 3:**
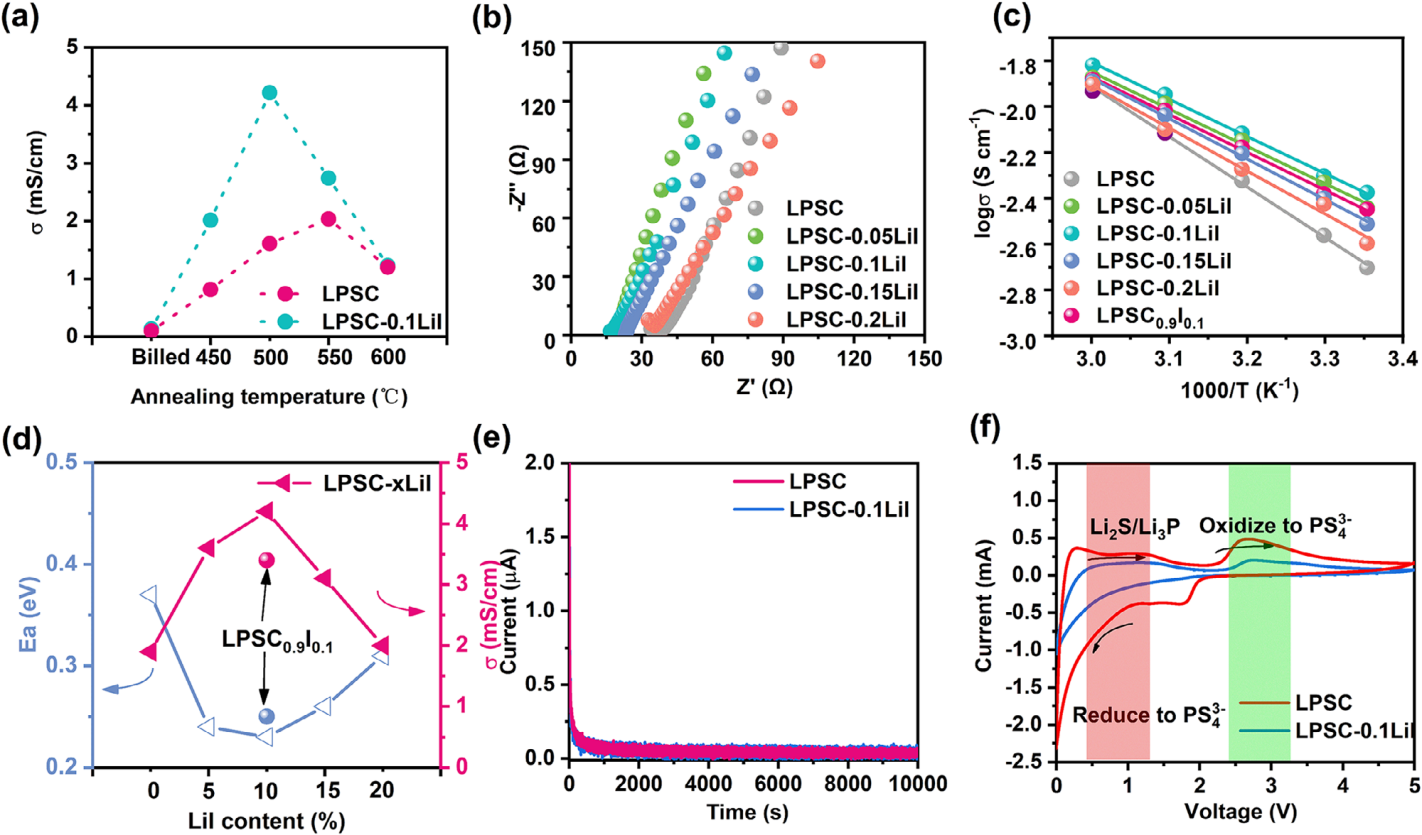
(a) The ionic conductivity of the pelletized samples sintered at 450°C, 500°C, 550°C, and 600°C. (b) The Nyquist plots of the LPSC‐*x*LiI (*x* = 0, 0.05, 0.1, 0.15, and 0.2) sintered at 500°C. (c) The Arrhenius plots of the LPSC‐*x*LiI (*x* = 0, 0.05, 0.1, 0.15, and 0.2) and LPSC_0.9_I_0.1_ sintered at 500°C, measured at a temperature range of 298–333 K. (d) Ionic conductivity and activation energy variation with varied LiI levels. (e) DC polarization curves and (f) cyclic voltammetry tests of LPSC and LPSC‐0.1LiI.

According to previous literatures [22, 37, 38], the optimal annealing temperature for LPSC electrolytes is 550°C. However, the iodine‐doped LPSC with different ratios exhibited partial melting under the 550°C annealing process. To gain the optimal SSEs, the annealing temperature ranges from 450°C to 600°C was performed for all samples as demonstrated in Figure S7, in which the morphologies of SSEs will undergo significant transformation with the increase of I doping concentration and temperature. Subsequently, the electrochemical impedance spectroscopy (EIS) was measured to explore the ionic conductivity (*σ*) as shown in Figure 3a. The optimal ionic conductivity was achieved after annealing at 550°C, which is consistent with the optimal temperature reported in most literature for LPSC. It is noteworthy that the LPSC‐0.1LiI electrolyte exhibits the highest ionic conductivity under 500°C, which agrees with the phenomenon in chloride‐rich Li_4.5_PS_4.5_Cl_1.5_ electrolyte [26]. The increased annealing temperature results in decreased conductivity, likely because the increased I content reduced the crystallization temperature of the electrolyte, as evidenced by the electrolyte melting phenomenon in Figure S7. To further investigate the effect of annealing temperature on the structural stability of the electrolyte, Raman spectroscopy was characterized as depicted in Figure S8, where the characteristic peaks of the LPSC‐0.1LiI electrolyte at 500°C align well with typical argyrodite Raman signatures. As the temperature increased, the characteristic peaks of the PS_4_^3−^ tetrahedron significantly changed, in which the intensity of the main peaks at 429 cm^−1^ decreased, and the peaks at 196 and 266 cm^−1^ disappeared by 600°C. Meanwhile, the intensities of the peaks at 575 and 601 cm^−1^ surpass that of the main peak. These findings suggest that the decrease in conductivity at high temperatures may be caused by the destruction of the structure of PS_4_^3−^ tetrahedron. Therefore, the I‐doped electrolytes were synthesized using an annealing temperature of 500°C. Furthermore, the impedance spectra of electrolytes with varying LiI concentrations post‐annealing are presented in Figure 3b, and the Arrhenius curves over the temperature range of 303–333 K are depicted in Figure 3c. The ionic conductivity and activation energy were computed, and the variations in their relationship are depicted in Figure 3d. Compared to the other electrolytes, the LPSC‐0.1LiI electrolyte delivered the highest ion conductivity (4.34 mS cm^−1^) and the lowest activation barrier (0.25 eV), indicating the rapid movement of Li^+^ in LPSC‐0.1LiI. Furthermore, the LPSC‐0.1LiI electrolyte exhibits an ionic conductivity of 0.34 mS cm^−1^, even at a low temperature of −20°C in Figure S9. The excellent ionic conductivity can be attributed to the I‐doping, which expanded the lithium ion transport channel, reduced the electrostatic attraction to the electrolyte framework, and boosted the system disorder. Excess iodine, however, would impede the lithium network, which is consistent with the results of AIMD simulations. Then, the electronic conductivities (*σ*_ele_) of LPSC and LPSC‐0.1LiI electrolytes were carried out through DC polarization method and calculated based on the formula (*σ*_ele_ *= L / RS*) as depicted in Figure 3e, where the introduction of LiI has little effect on the electronic conductivities of the electrolytes. To demonstrate the improved decomposition voltage of solid electrolytes, the cyclic voltammetry (CV) curves of semi‐cells (Li/SSEs/C+SE) using LPSC and LPSC‐0.1LiI electrolytes were measured as shown in Figure 3f. The addition of carbon (C) fulfills the crucial function of promoting the kinetics of oxidation/reduction, bringing the measured value closer to the real window. It can be seen that the oxidation peak of the Li/LPSC‐0.1LiI/C+LPSC‐0.1LiI cell has elevated to around 2.6 V (vs. Li/Li^+^). Moreover, the reduction currents were observed in two samples, which are associated with lithium‐containing by‐products like Li_3_P, Li_2_S, and LiCl [39]. The formation of these by‐products results from reactions between the electrolyte and lithium metal at voltages below 0.5 V, which might inhibit the transport kinetics of Li^+^ and lead to the inhomogeneous deposition of the lithium metal, subsequently causing the uncontrolled growth of lithium dendrites [40]. But the peak currents of LPSC‐0.1LiI electrolyte were smaller than those of LPSC, indicating enhanced redox stability towards lithium metal and suppression of lithium‐metal reduction at the interface.

### 2.4 Interfacial Compatibility of SSEs Against Metallic Li

**FIGURE 4:**
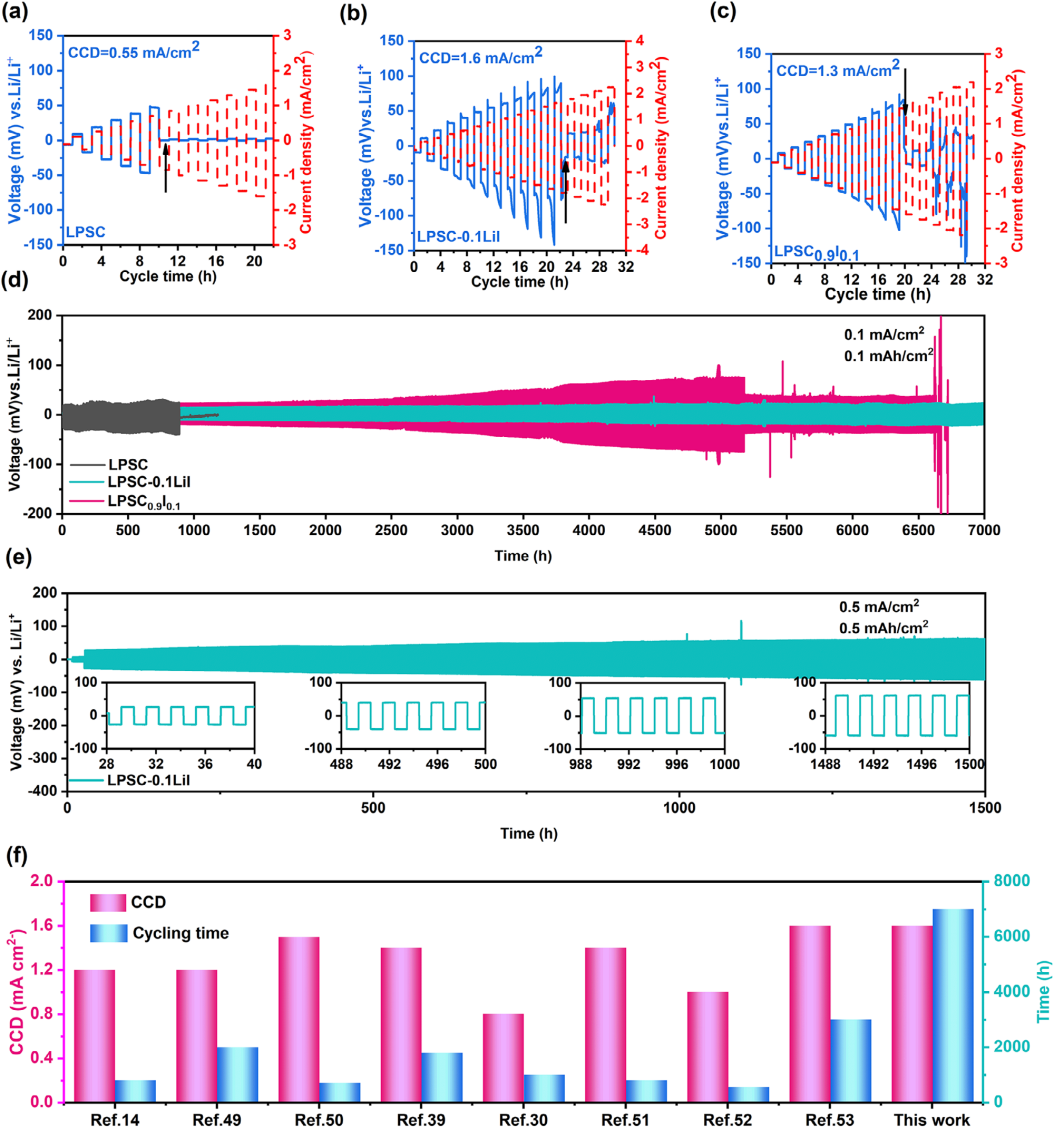
Galvanostatic cycling of the Li symmetric cells of (a) LPSC, (b) LPSC‐0.1LiI, and (c) LPSC_0.9_I_0.1_ at step‐increased current densities at 30°C. Galvanostatic cycling of the Li symmetric cells with LPSC‐0.1LiI electrolyte at (d) 0.1 mA cm^−2^/0.1mAh cm^−2^ and (e) 0.5 mA cm^−2^/0.5mAh cm^−2^. (f) Summary of reported critical current densities and cycling time of Li–Li symmetric cell.

Due to the electrochemically incompatible problem between the sulfide electrolytes and Li metal, the critical current density (CCD) is usually less than 1 mA cm^−2^ and the cycling life of Li/LPSC/Li battery is always less than 1000 h at a current density of 0.1 mA cm^−2^ [11, 14, 15]. To assess the capability of the prepared LPSC‐*x*LiI electrolytes in resisting lithium dendrite growth, the CCD and cycling life of Li symmetric cells were measured at room temperature. As is shown in Figure 4a,b and Figure S10, the voltage of Li/LPSC/Li symmetrical cell suffers an irreversible drop as the current density gradually elevates to 0.55 mA cm^−2^, implying that an internal short circuit occurs in the cell due to the uncontrollable Li dendrite growth. The CCD value of Li symmetric cells with LPSC‐*x*LiI electrolytes increases and attains the highest value of 1.6 mA cm^−2^ when *x* is equal to 0.1. With the increase in I content, the CCD value of the electrolyte subsequently decreased due to the reduction in ionic conductivity of the electrolytes [41, 42]. In addition, the CCD value (1.3 mA cm^−2^) of the LPSC_0.9_I_0.1_ electrolyte is also significantly higher than that of the LPSC electrolyte (Figure 4c), indicating that the introduction of I has an inhibitory effect on lithium dendrite growth. Interestingly, although the ionic conductivity of LPSC_0.9_I_0.1_ is lower than that of LPSC‐0.05LiI (Figure S10a), it exhibits a higher CCD than LPSC‐0.05LiI, implying that ionic conductivity is not the only determinant of CCD values. It might arise from the tuning of interfacial chemistry after incorporating iodine into the electrolyte, which positively affects the construction of the SEI layer, improves Li plating/stripping behavior, and enhances the ability to inhibit dendrite growth [43, 44].

Moreover, the durability and lifelong cycle stability with Li metal were further evaluated by galvanostatic charge/discharge measurements at 30°C under various current densities and cutoff capacities. The voltage curves of Li/LPSC/Li symmetrical cell were characterized at a current density of 0.1 mA cm^−2^ and a cut‐off capacity of 0.1 mAh cm^−2^, as presented in Figure 4d. A sudden overpotential drop appeared after cycling 890 h due to the soft short circuit, which was caused by the formation of an unstable interface after the reaction between LPSC and lithium metal, leading to uneven lithium deposition and ultimately lithium dendrite penetration. But the Li/LPSC‐0.1LiI/Li symmetrical cell achieved a stable cycling time for 7000 h, with the polarization voltage increasing only from 8 to 18 mV, implying superior stability toward the bare lithium metal. As a comparison, the Li symmetrical cells of Li/LPSC‐0.05LiI/Li, Li/LPSC‐0.15LiI/Li, and Li/LPSC‐0.2LiI/Li were measured and presented in FiguresS11–S13, in which the overpotential and cycle stability were significantly improved after I‐doped electrolytes. However, the cycling performances of these Li symmetrical cells are worse than those of Li/LPSC‐0.1LiI/Li cells due to low ionic conductivity and thicker iodinated interphase formed during plating/stripping. Especially, when comparing the two electrolytes with substituted Cl sites (LPSC_0.9_I_0.1_) and substituted S sites (LPSC‐0.1LiI), the Li/LPSC‐0.1LiI/Li cell exhibits more excellent performance due to the high degrees of X^−^/S^2−^ disorder. Additionally, the Li/LPSC‐0.1LiI/Li cell was cycled for 20 h (10 cycles) at 0.1 mA cm^−2^ to form a more stable interfacial layer and then tested under 0.5 mA cm^−2^ and 0.5 mAh cm^−2^ as shown in Figure 4e. The Li/LPSC‐0.1LiI/Li cell delivered stable cycling for 1500 h, with the overpotential increasing slowly from 27 to 58 mV without short circuit, demonstrating the excellent Li compatibility of LPSC‐0.1LiI electrolyte. Furthermore, the performances of Li/LPSC‐0.1LiI/Li cells were comparable to those of various sulfide‐based lithium symmetric cells, as depicted in Figure 4f and Table S4 [14, 30, 39, 45, 46, 47, 48, 49].

**FIGURE 5:**
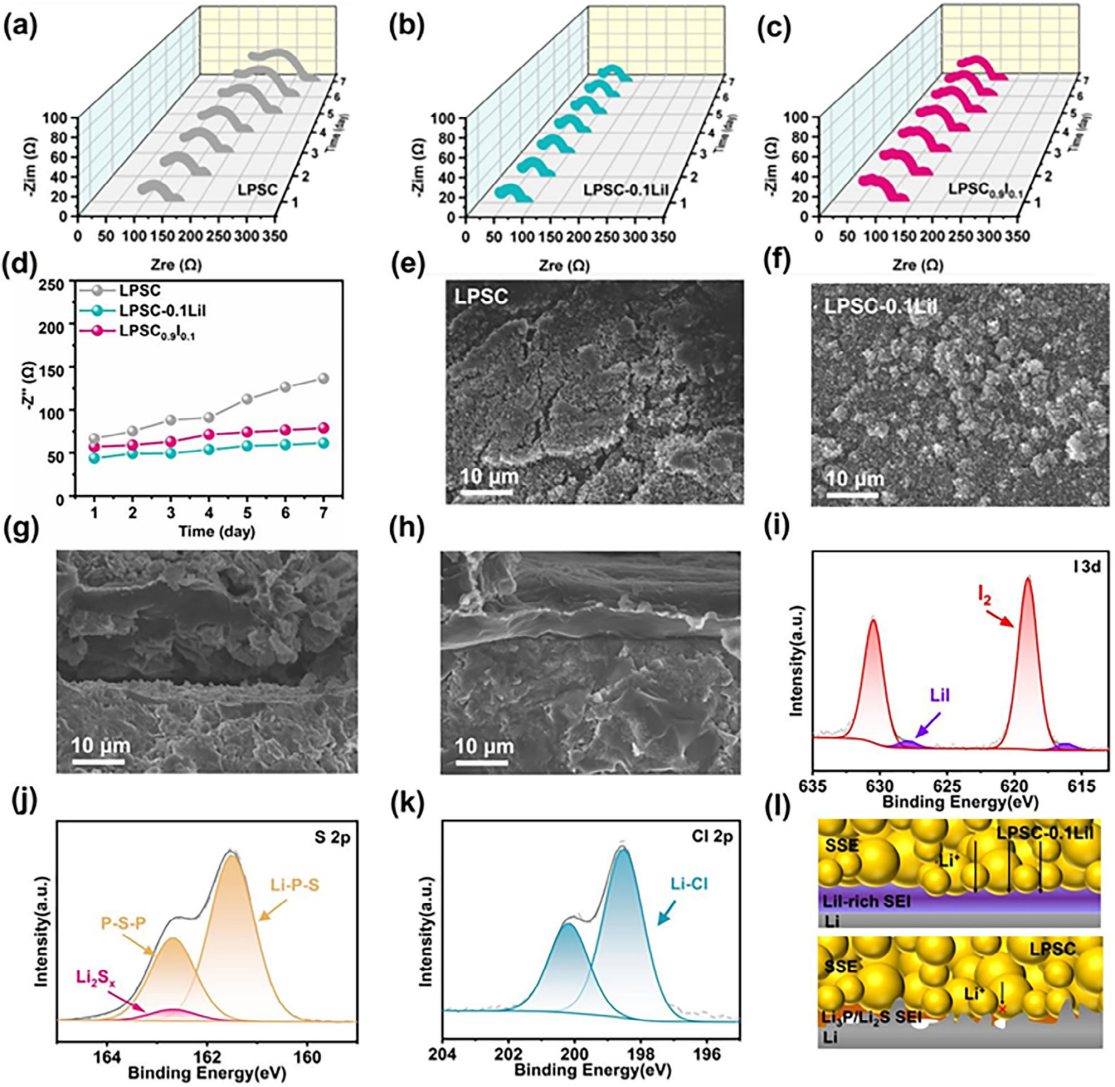
Time‐resolved EIS spectra of Li symmetric cells up to 300 h using (a) LPSC, (b) LPSC‐0.1LiI, and (c) LPSC_0.9_I_0.1_. (d) *R*_SEI_ value change curves at different standing aging times. SEM images of the surface topography of Li symmetric cells using (e) LPSC and (f) LPSC‐0.1LiI. SEM images of the cross‐section of Li symmetric cells using (g) LPSC and (h) LPSC‐0.1LiI. XPS measurement on the Li‐metal side after the fifth cycle (i) I, (j) S, and (k) Cl, respectively. (l) Schematic diagram of Li plating on the (top) LPSC‐0.1LiI and (down) LPSC interface evolution.

To understand the reason behind the high performance of Li/LPSC‐0.1LiI/Li symmetric cells, Figure 5a–c shows time‐resolved EIS changes of symmetric batteries assembled with LPSC, LPSC‐0.1LiI, and LPSC_0.9_I_0.1_ electrolytes in the open‐circuit state within 7 days, respectively. The impedance values of Li symmetric cells with LPSC‐0.1LiI are the lowest and most steady compared to those of Li symmetric cells with LPSC and LPSC_0.9_I_0.1_ electrolytes, indicating the low decomposition and few porous Li interfaces during the cycling process [43]. Moreover, the detailed interfacial charge transfer impedance values of each component were obtained by fitting based on the equivalent circuit diagram (Figure S14) as shown in Figure 5d, in which the initial impedance value of Li/LPSC‐0.1LiI/Li (43 Ω) is lower than those of Li/LPSC_0.9_I_0.1_/Li (58 Ω) and Li/LPSC/Li (65 Ω), indicating high ionic conductivity and superior electrolyte interface in LPSC‐0.1LiI. As lithium plating and stripping proceed, side reactions and contact changes occur at the interface between the electrolyte and lithium metal, leading to an increase in interfacial impedance over time. For the LPSC sample, the impedance value rapidly increased to 130 Ω after 7 days because of uneven lithium deposition on the LPSC/Li interface and void formation, resulting in poor interfacial contact, high interfacial resistance. In contrast, the LPSC‐0.1LiI electrolyte exhibits the lowest impedance value and the smallest change, indicating its superior interfacial stability and compatibility with lithium metal.

In order to further investigate the effect of I doping on the lithium stripping/deposition behavior, characterizing technologies are of critical importance in enabling the revealing of materials regarding their structural and chemical information [44, 50, 51]. Therefore, the lithium symmetric cells were disassembled after 300 h at 0.1 mA cm^−2^. The SEM images of the surface and cross‐section topography of LPSC electrolyte and LPSC‐LiI electrolyte are presented in Figure 5e–h, where the LPSC‐LiI has obviously a smoother surface than that of LPSC, and no obvious localized cracks on the surface (Figure 5e,f), indicating the excellent interfacial chemical stability. From the cross‐sectional SEM images (Figure 5g), a separated interface can be found between the electrolyte and lithium metal after cycling in Li symmetric cell with LPSC. This separation may result in a difference between lithium stripping /deposition, leading to increased internal resistance [43, 45, 52, 53]. Impressively, a consistently close contact between the LPSC‐0.1LiI and lithium anode is maintained throughout the entire cycling process due to uniform deposition of lithium metal at the interface. To investigate the influence of I doping on interface compatibility more comprehensively, XPS tests were carried out to characterize the chemical composition of the Li/LPSC‐0.1LiI interface after cycling. Figure 5i–k and Figure S15 present the XPS spectra of I 3d, S 2p, Cl 2p, P 2p, and Li 1s at the Li/LPSC‐0.1LiI interface. After cycling, a small number of decomposed products consisting of Li‐I alloy and LiCl are found to be generated at the interface. The binding energy of each component is consistent with other previous literature reports. The LiI‐rich SEI with high interfacial energy can also play a critical role in relieving the decomposition of the electrolyte. It can effectively stabilize and reduce the resistance of the Li/SSEs interface. Therefore, as shown in the schematic diagram in Figure 5l, the fast ionic transport and stable SEI will take place in ASSBs with LPSC‐0.1LiI and LPSC_0.9_I_0.1_ electrolytes, which prevents the formation of voids and dendrites.

### 2.5 Full Battery Performance

**FIGURE 6:**
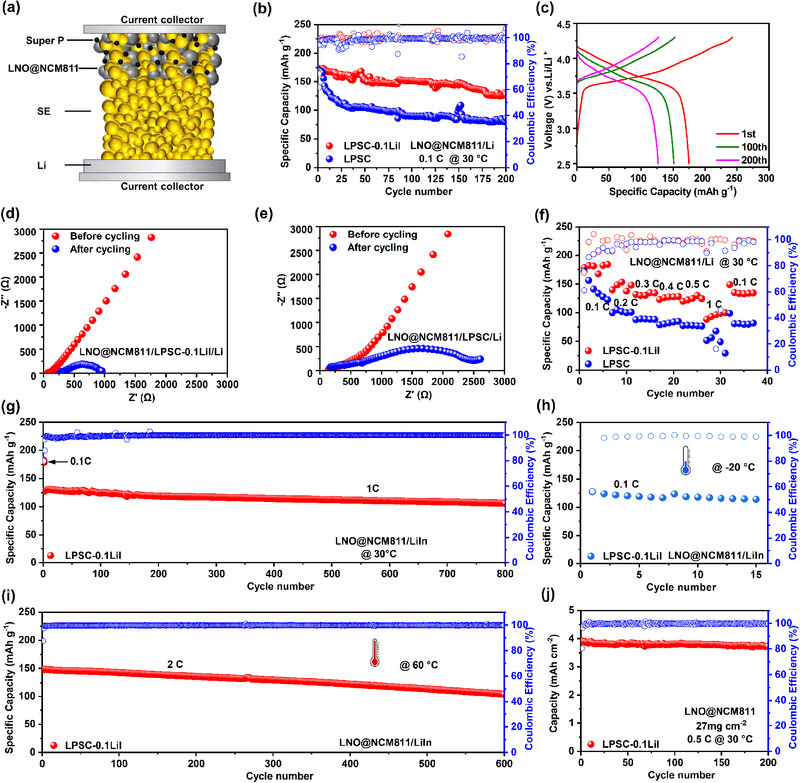
(a) Schematic diagram of the LNO@NCM811/SEs/Li cell. (b) Cyclic performance and (c) charge–discharge voltage curves of LNO@NCM811/LPSC/Li cell and LNO@NCM811/LPSC‐0.1LiI/Li cell at 0.1C at 30°C. Nyquist plots before and after cycling of (d) LNO@NCM811/LPSC‐0.1LiI/Li cell and (e) LNO@NCM811/LPSC/Li cell. (f) Rate capability of LNO@NCM811/LPSC/Li and LNO@NCM811/LPSC‐0.1LiI/Li cells at 30°C. (g) Cyclic performance of LNO@NCM811/LPSC‐0.1LiI/LiIn cell at 1C at 30°C. (h) Cyclic performance at low temperatures (−20°C) of LNO@NCM811/LPSC‐0.1LiI/LiIn cell at 0.1C. (i) Cyclic performance at high temperatures (60°C) of LNO@NCM811/LPSC‐0.1LiI/LiIn cell at 2C. (j) Cyclic performance of high loading LNO@NCM811/LPSC‐0.1LiI/LiIn cell at 0.5C at 30°C.

To demonstrate the application of the LPSC‐0.1LiI electrolyte in ASSBs, the LPSC‐0.1LiI electrolyte was first combined with LiNbO_3_‐coated LiNi_0.8_Mn_0.1_Co_0.1_O_2_ (LNO@NCM811) cathode material to assemble ASSBs. The schematic diagram of LNO@NCM811/LPSC‐0.1LiI/Li(LiIn) configuration is described in Figure 6a. The cycling performance of ASSBs with active materials loading of 6.3 mg cm^−2^ was tested from 2.5 to 4.3 V at a 0.1C rate. As shown in Figure 6b,c, the LNO@NCM811/LPSC‐0.1LiI/Li delivers an initial discharge capacity of 174.8 mAh g^−1^ with a high capacity retention of 72.6% after 200 cycles. Notably, for the ASSBs using LPSC as the electrolyte layer (LNO@NCM/LPSC/Li), their capacity sharply decreases from 171.1 to 86.1 mAh g^−1^ after 200 cycles at 0.1C with only 50.3% capacity retention. As presented in Figure 6d, due to low internal resistance and rapid kinetics, the interface resistance of LNO@NCM/LPSC‐0.1LiI /Li remained almost unchanged before and after cycling. This further indicates that the stability and compatibility of the interface are enhanced during long‐term cycling. On the contrary, the impedance resistance of the ASSBs with LPSC increased significantly after cycling, as shown in Figure 6e. The rate performances of ASSBs were also evaluated in Figure 6f, in which the ASSLBs with LPSC‐0.1LiI achieved capacities of 178.8, 139.9, 132, 123.1, 120, and 88.5 mAh g^−1^ at 0.1C, 0.2C, 0.3C, 0.4C, 0.5C, and 1C, respectively. After the current density returns to 0.1C, the ASSBs still showcase a high discharging capacity of 149 mAh g^−1^. Conversely, due to the poor ionic conduction and interface stability, ASSBs with LPSC generally show low capacities and inferior rate performance.

On the other hand, using Li‐In as anode, LNO@NCM811/LPSC‐0.1LiI/Li‐In cell operated at 1C delivered an initial discharge capacity of 127.8 mAh g^−1^ and 83.2% capacity retention after 800 cycles (Figure 6g and Figure S16). Afterward, the cells also exhibited a discharge capacity of 125 mAh g^−1^ under −20°C because of the favorable ion transport capacity of the electrolyte (Figure 6h and Figure S17). Meanwhile, the discharge capacity reached 147.4 mAh g^−1^ and still remained at 104.2 mAh g^−1^ after 600 cycles at 2 C under 60°C (Figure 6i). High ionic conductivity and excellent interface stability provide the possibility for the cell with LPSC‐0.1LiI with a high areal mass loading of 27 mg cm^−2^ of active materials. Figure 6j and Figure S18 display a high reversible capacity of 3.89 mAh cm^−2^ and excellent cycling stability with 96.9% capacity retention after 200 cycles and with Coulombic efficiency of higher than 99% at 0.5C under 30°C. Furthermore, to prove the compatibility of LPSC‐0.1LiI electrolyte with other cathodes, ASSBs were assembled using LiCoO_2_ (LCO) and S cathodes with Li‐In anode as depicted in Figures S19–S21. The LCO/LPSC‐0.1LiI/LiIn cell delivered an initial discharge specific capacity of 113.3 mAh g^−1^ and Coulombic efficiency of 87.2% at 0.1C. After the gradual activation of the active material sulfur, the S/LPSC‐0.1LiI/LiIn exhibited a high reversible capacity of 1215.9 mAh g^−1^ at 1C under 30°C. These results further demonstrate the excellent suitability of electrolytes for practical applications.

## 3 Conclusion

In summary, AMID calculations were used to investigate the principles of ion transport and the impact of halogen diversity on argyrodite, and screening electrolytes with high ionic conductivity, revealing that using iodine substitution at the sulfur site (4d) is more favorable to accelerate lithium‐ion migration with a low migration barrier. Then, the Li_6−_ *_x_* PS_5−_ *_x_* ClI*_x_* (*x* = 0, 0.05, 0.1, 0.15, and 0.2) argyrodites were successfully synthesized and yielded a high degree of X^−^/S^2−^ disorder, in which the highest room‐temperature ionic conductivity of 4.2 mS/cm associated with the lowest activation barrier of 0.25 eV was found in LPSC‐0.1LiI. Furthermore, the development of a robust interface significantly enhances cycle stability, achieving up to 7000 h at 0.1 mA h cm^−2^ and 1500 h at 0.5 mA h cm^−2^ in lithium symmetrical cells. The full cells, composed of various cathodes (LNO@NMC811, LCO, and S), Li or Li‐In anode, and LPSC‐0.1LiI electrolyte, exhibit excellent electrochemical performance, further demonstrating their practicality. This work advances the understanding of halogen occupancy in argyrodite materials, particularly concerning ion transport and electrochemical stability. Furthermore, it provides an effective approach to addressing solid interfacial challenges in high‐performance ASSBs.

## Conflicts of Interest

The authors declare no conflicts of interest.

## Supporting information




**Supporting Information file 1**: exp270082‐sup‐0001‐SuppMat.docx

## Data Availability

The data that support the findings of this study are available from the corresponding author upon reasonable request.
