# Supplementary material for: Elucidating and Optimizing I Occupation in Lithium Argyrodite Solid Electrolytes for Advanced All‐Solid‐State Li Metal Batteries
Source: Exploration (Beijing). 2025 Aug 25;5(5):20240050. doi: 10.1002/EXP.20240050 (PMC12561285; doi:10.1002/EXP.20240050)
Supplement: Supplementary file 1 — Supporting Information file 1: exp270082‐sup‐0001‐SuppMat.docx [file EXP2-5-20240050-s001.docx]

**Elucidating and Optimizing I occupation in Lithium Argyrodite Solid Electrolytes for Advanced All-Solid-State Li Metal Batteries**

Zhikai Huang^a^, Wenrui Sun^a^, Shuaiyu He^a^, Huan Hu^a^, Xue Li^a^, Ke Huang^a^, Zhihao Yan^a^, Gencai Guo^b^, Yaojie Lei^d^, Liwen Yang^b^, Jianyu Huang^a^, Gang Wang^a^, Yaru Liang^a^^[[1]](#footnote-1)^*, Guobao Xu^a^^[[2]](#footnote-2)^*, Xingqiao Wu^c^^[[3]](#footnote-3)^*

*^a^**Hunan Provincial Key laboratory of Thin Film Materials and Devices, School of Material Sciences and Engineering, Xiangtan University, Xiangtan, 411105, China*

*^b^School of Physics and Optoelectronics, Xiangtan University, Xiangtan, 411105, China*

*^c^Institute for Carbon Neutralization Technology, College of Chemistry and Materials Engineering, Wenzhou University, Wenzhou, 325035, China*

*^d^Centre for Clean Energy Technology, University of Technology Sydney, Sydney, NSW 2007*

**Experimental Section**

**Synthesis of electrolyte**

The halogen-rich Li_6-x_PS_5-x_ClI_x_(x = 0,0.05, 0.1, 0.15, and 0.2) sulfide solid electrolytes, Li_2_S (99.9%, Sigma-Aldrich), P_2_S_5_ (99%, Sigma-Aldrich), LiCl (99%, Macklin), LiI (99.9%, Macklin) were weighed based on an appropriate stoichiometric ratio, the mixture was premixed using the planetary ball-milled equipment at 180 rpm min^-1^ for 1 h then high-energy ball-milled at 450 rpm min^-1^ and 30 h respectively. The precursor powder was pelletized and sealed in a quartz tube and sintered between 450 and 600 °C for 5 h with a heating rate of 5 °C min^-1^ followed by naturally cooling down. The final solid electrolyte was obtained after hand grinding for 30 minutes. All the above steps were carried out in glove boxes under argon atmosphere protection.

**Fabrication of the cathode composite**

The LNO@NCM811 and LCO cathode composites were prepared by hand grinding for 30 min using LiNbO_3_-coated LiNi_0.8_Co_0.1_Mn_0.1_O_2_ or LiCoO_2_, solid electrolyte and Super P (mass ratio 70:30:3). The sulfur-carbon(S/C) was prepared by mixing elemental sulfur (S), expanded graphite (EG), with a mass ratio of S/EG =3:1, and consequent heating at 300 °C for 10 h. The S cathode composite was prepared by high-energy ball milling the S/EG composite, single-walled carbon nanofibers(sCNF) and SSE, with a mass ratio of 8:2:1 at 400 rpm min^-1^ for 20 h.

**Materials characterizations**

The XRD (Rigaku SmartLab) using Cu Kα radiation was recorded at the scanning rate of 5 s per step from 2θ of 10°-90°. Electrolyte powders were kept in with a polyimide film to avoid air. Raman spectra were collected from Renishaw InVia system under a laser beam excitation source with 532 nm, and acquisition time of 10 s. The samples were characterized by XPS (Kratos Analytical Ltd., UK) using an Al Kα source, the operating voltage is 15kV and the filament current is 10 mA, the passing energy was 50 eV, the step size was 0.05 eV, the number of scans was 5, and the X-ray spot size was 100 μm × 100 μm. The obtained binding energies were calibrated with the C ls peak at 284.8 eV. The accelerating voltage of scanning electron microscopy (SEM, ZEISS Merlin Compact) is 20kV, the imaging modes used was SE, and the magnification range was 1000 to 5000 times. High-angle annular dark-field scanning transmission electron microscopy (HAADF-STEM) and corresponding EDS measurements were carried out in a TEM (Titan cubed Themis Z) at 300 kV. The collection semi-angle of the STEM detectors was set to 41-200 mrad for HAADF imaging. The TEM grid was loaded onto the cryo-holder in an Ar atmosphere and quickly transferred to the STEM at cryogenic temperature. These specimens were prepared in an Ar-filled glove box and transported with air-tight sample holders (O_2_, H_2_O < 0.01 ppm).

**Electrochemical characterizations**

Electrochemical impedance spectroscopy (EIS) was employed to test the ionic conductivity of the solid electrolyte using an electrochemical workstation (Donghua Analysis, DH7001) in the frequency range from 0.1 Hz to 1 MHz, and the electrolyte powders were cold-pressed into pellets with the thickness of 560 microns and a diameter of 10 mm under the pressure of 250 MPa. The stainless steel(SS) collector was used as the blocking electrode. Direct current(DC) polarization method was carried out to analyze the electronic conductivity of electrolytes based on SS/SEs/SS cells. Cyclic voltammetry (CV) test in the voltage range 0 to 5V at a scanning speed of 5 mV S^-1^ on C+SSEs/SSEs/Li cell.

**Assembling process of solid-state battery**

All-solid-state batteries were assembled in a PEEK mold with a diameter of 10 mm, 100 mg of electrolyte was first cold-pressed into a round sheet under 200 MPa pressure. Then, 6-7 mg of cathode composite was evenly dispersed on one side of SSE and pressed under 300 MPa, and then Li (99.9% purity, 100μm) or LiIn foil was attached on the other side of the electrolyte. Finally, SS column was used as the current collector. Constant-current charge-discharge was carried out on the LAND battery test system (CT-2001A, Wuhan Rambo Testing Equipment Co., Ltd.). The voltage window of LNO@NCM811/SEs/Li cells was set to 2.5-4.3 V (vs. Li/Li^+^), the voltage window of LNO@NCM811/SEs/LiIn and LCO/SE/LiIn cells was set to 2.1-3.8 V (vs. Li/LiIn), the voltage window of S/SE/LiIn cells was set to 0-3 V (vs. Li/LiIn). All battery manufacturing processes were carried out in an argon-filled glove box (O_2_, H_2_O < 0.01 ppm).

**Calculation Details:**

All ab initio molecular dynamics (AIMD) simulations in this work were performed using the Vienna Ab initio Simulation Package(VASP) and Projected Augmented Wave (PAW) pseudopotentials. The exchange and correlation energies were calculated using the Perdew, Burke, and Ernzerhof (PBE) functional within the generalized gradient approximation (GGA). The calculated parameters are similar to those used in the Materials Project (MP). All analyses were performed using the Pymatgen and Pymatgen-diffusion packages, and the initial structure of Li_6_PS_5_Cl was obtained from the MP database (ID: MP-985592). The diffusion of lithium ions in the NVT system was investigated using non-spin-polarized AIMD simulations at room temperature in the temperature range of 300 to 1200 K in increments of 300 K. The DFT method for structure optimization was applied to the AIMD simulations to maintain the minimum lattice dimension of the super-cell larger than 10 Å, with a plane-wave energy cutoff of 300 eV, and the use of a Γ-centered 1×1× 1 k-mesh. All AIMD calculations were performed using an automated in-house AIMD workflow program with a time step of 1 fs for a total of 50 ps. All AIMD calculations were performed using an automated in-house AIMD workflow program with a time step of 1 fs for a total of 50 ps. The diffusion coefficients were calculated based on the mean-square displacements (MSDs) over the temperature range from 300 to 1200 K. Subsequently, a convergent Arrhenius plot was fitted to find the activation energy (Ea).





**Figure S1** XRD patterns of LPSC-xLiI electrolytes





**Figure S2** Raman spectra of LPSC-xLiI electrolytes





**Figure S3** Rietveld refinements and structure diagram of LPSC electrolyte





**Figure S4** XRD and Raman patterns of LPSC_0.9_I_0.1_ electrolyte





**Figure S5** Rietveld refinements and structure diagram of LPSC_0.9_I_0.1_ electrolyte





**Figure S6 .** XPS measurement on the LPSC-0.1LiI (a) S 2p spectra, (b) P 2p spectra, (c) Cl 2p spectra, (d) Li 1s spectra and LPSC (e) S 2p spectra, (f) P 2p spectra, (g) Cl 2p spectra, (h) Li 1s spectra, respectively.


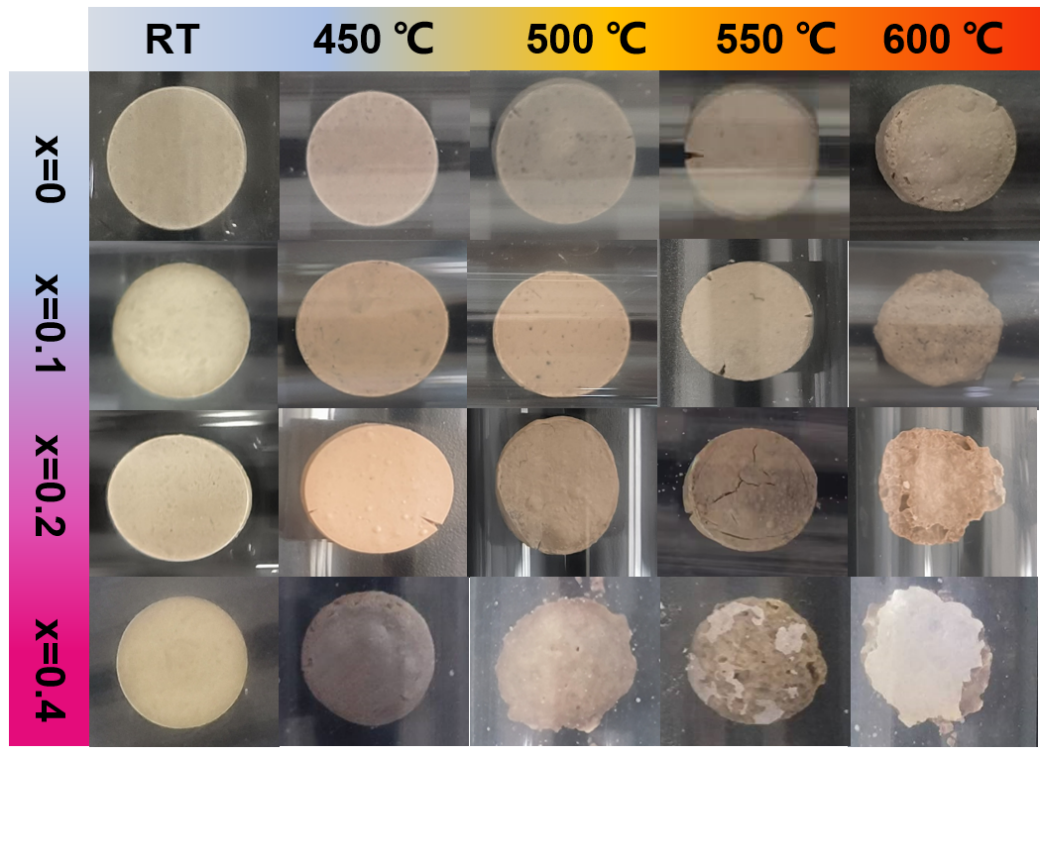


**Figure S7** Optical photographs of LPSC-xLiI electrolytes after annealing at different temperatures (450-600 ℃)





**Figure S8** Raman spectral of LPSC-0.1LiI electrolyte at different annealing temperatures

*
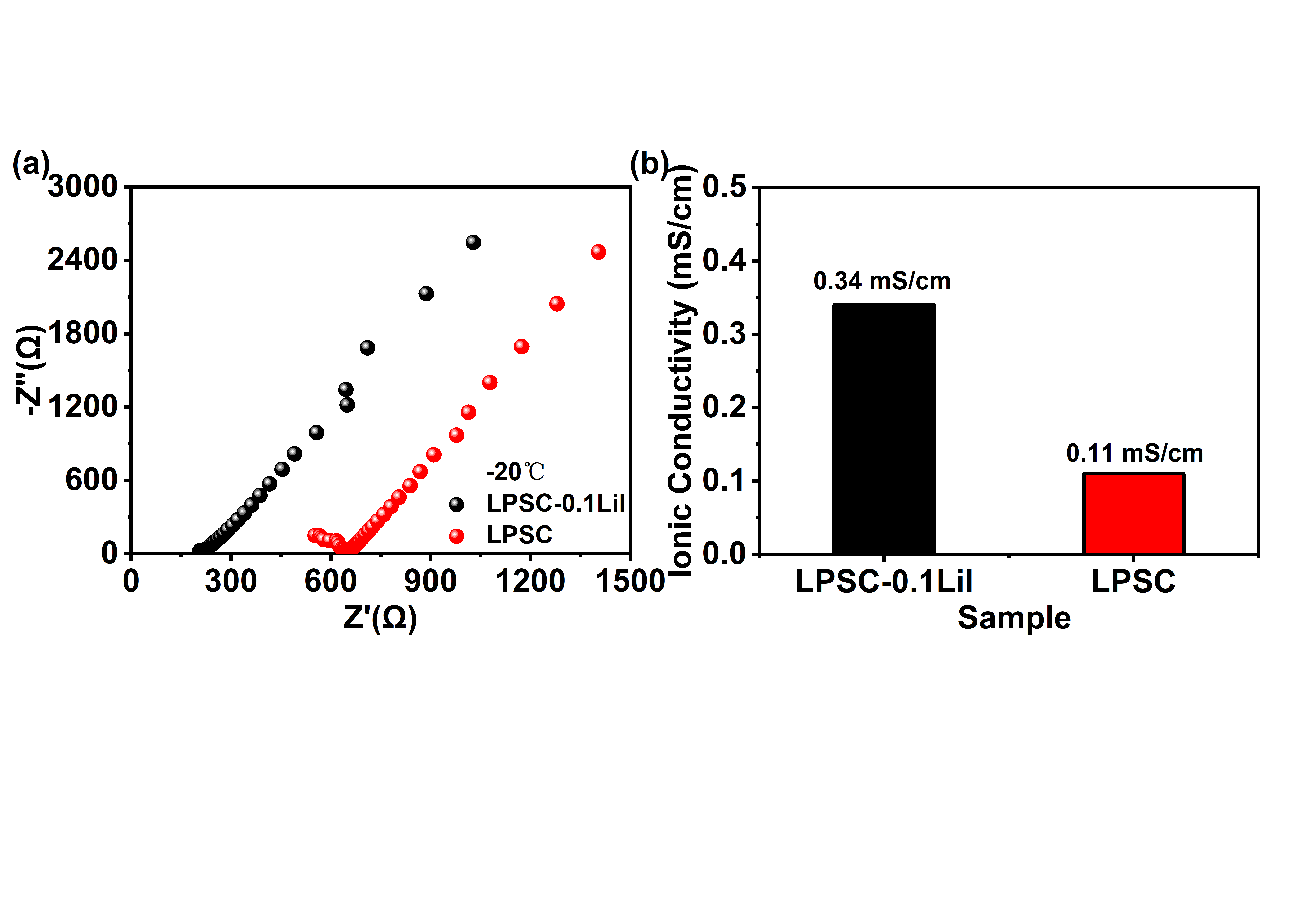
*

**Figure S9** (a) Nyquist plot of LPSC and LPSC-0.1LiI at -20℃and (b) Ionic conductivity of LPSC and LPSC-0.1LiI at -20℃.





**Figure S10** Galvanostatic cycling of the Li symmetric cells of (a) LPSC-0.05LiI, (b) LPSC-0.15LiI and (c) LPSC-0.2LiI at step-increased current densities at 30 ℃.





**Figure S11** Galvanostatic cycling of the Li symmetric cells with LPSC-0.05LiI electrolyte at 0.1mA cm^-2^/0.1mAh cm^-2^





**Figure S12** Galvanostatic cycling of the Li symmetric cells with LPSC-0.15LiI electrolyte at 0.1mA cm^-2^/0.1mAh cm^-2^





**Figure S13** Galvanostatic cycling of the Li symmetric cells with LPSC-0.2LiI electrolyte at 0.1mA cm^-2^/0.1mAh cm^-2^


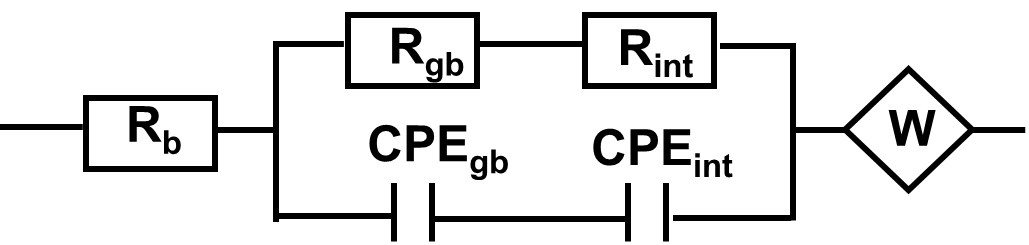


**Figure S14** The equivalent circuit for fitting the Nyquist plots. All impedance spectra were fitted by using an equivalent circuit of R_b_(R_gb_-CPE_gb_)(R_int_-CPE_int_)W, which consists of a bulk resistance (R_b_), a parallel unit (R_gb_-CPE_gb_) related to the grain boundary resistance of the argyrodite SE, a second parallel unit (R_int_-CPE_int_) related to the interfacial layer resistance, and a Warburg element (W).


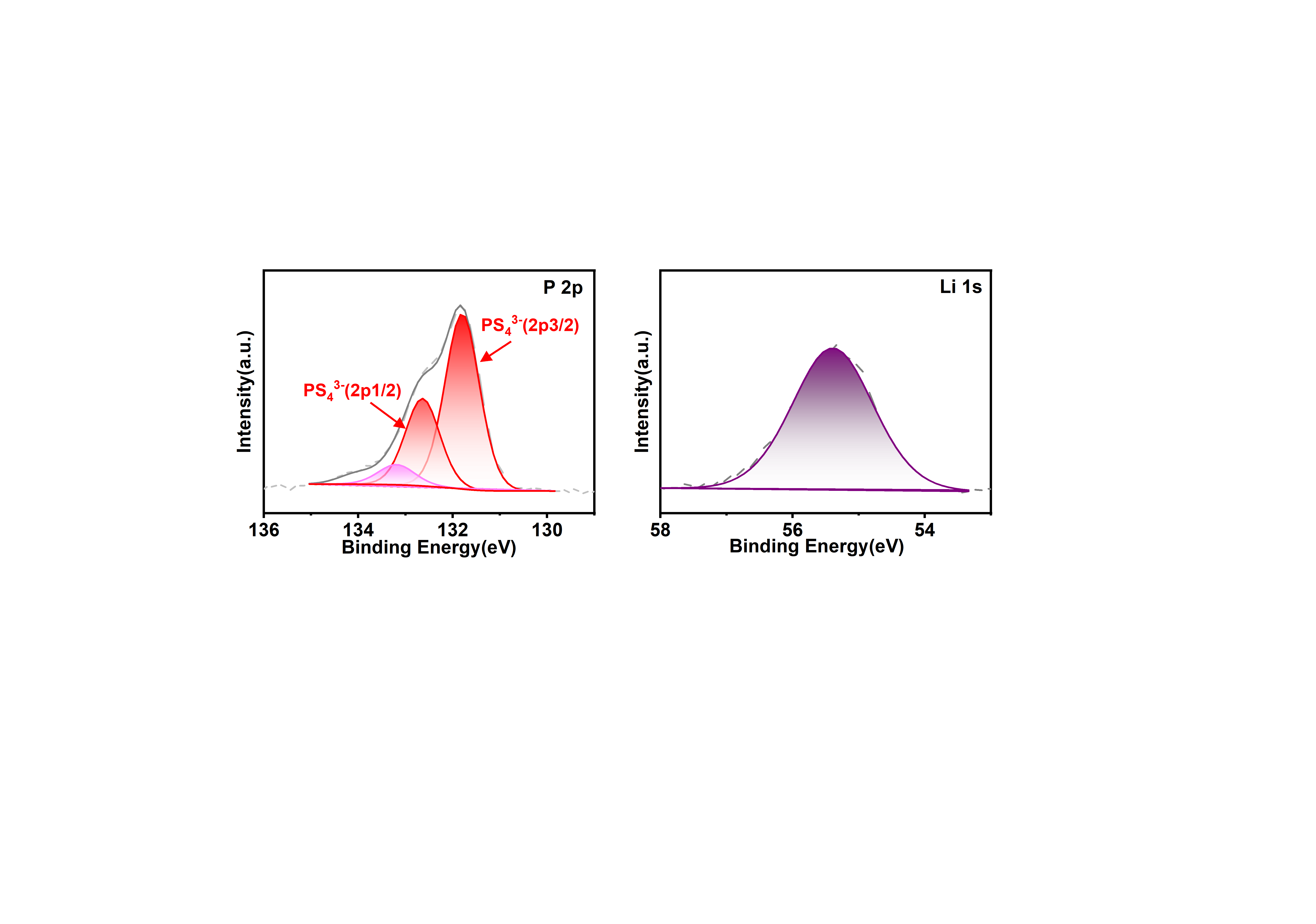


**(b)**

**(a)**

**Figure S15** XPS measurement on the Li-metal side after the 5th cycle (a) P 2p, (b) Li 1s.





**Figure S16** charge-discharge voltage curves of LNO@NCM811/LPSC-0.1LiI/LiIn cell at 1C at 30 ℃





**Figure S17** charge-discharge voltage curves at low temperatures (-20 ℃) of LNO@NCM811/LPSC-0.1LiI/LiIn cell at 0.1 C





**Figure S18** charge-discharge voltage curves of high loading LNO@NCM811/LPSC-0.1LiI/LiIn cell at 0.5 C at 30 ℃.





**Figure S19** Cyclic performance of LCO/LPSC-0.1LiI/LiIn cell at 0.1C at 30 ℃





**Figure S20** charge-discharge voltage curves of LCO/LPSC-0.1LiI/LiIn cell at 0.1C at 30 ℃





**Figure S21** Cyclic performance of S/LPSC-0.1LiI/LiIn cell at 1C at 30 ℃

**Table S1**. Crystallographic data of LPSC (space group = F-43m)

| **Atom** | **Wyckoff Site** | **x** | **y** | **z** | **Occ.** |
| --- | --- | --- | --- | --- | --- |
| **Li1** | 48h | 0.3203 | 0.0182 | 0.6798 | 0.5 |
| **Cl1** | 4a | 0 | 0 | 1 | 0.385 |
| **Cl2** | 4d | 0.25 | 0.25 | 0.75 | 0.615 |
| **P1** | 4b | 0 | 0 | 0.5 | 1 |
| **S1** | 4d | 0.25 | 0.25 | 0.75 | 0.385 |
| **S2** | 16e | 0.1186 | -0.1186 | 0.6186 | 1 |
| **S3** | 4a | 0 | 0 | 1 | 0.615 |

**Table S2**. Crystallographic data of LPSC-0.1LiI (space group = F-43m)

| **Atom** | **Wyckoff Site** | **x** | **y** | **z** | **Occ.** |
| --- | --- | --- | --- | --- | --- |
| **Li1** | 48h | 0.3203 | 0.0182 | 0.6798 | 0.495 |
| **Cl1** | 4a | 0 | 0 | 1 | 0.377 |
| **Cl2** | 4d | 0.25 | 0.25 | 0.75 | 0.623 |
| **P1** | 4b | 0 | 0 | 0.5 | 1 |
| **S1** | 4d | 0.25 | 0.25 | 0.75 | 0.277 |
| **S2** | 16e | 0.1186 | -0.1186 | 0.6186 | 1 |
| **S3** | 4a | 0 | 0 | 1 | 0.623 |
| **I1** | 4d | 0.25 | 0.25 | 0.75 | 0.1 |

**Table S3**. Crystallographic data of LPSC_0.9_I_0.1_ (space group = F-43m)

| **Atom** | **Wyckoff Site** | **x** | **y** | **z** | **Occ.** |
| --- | --- | --- | --- | --- | --- |
| **Li1** | 48h | 0.3203 | 0.0182 | 0.6798 | 0.5 |
| **Cl1** | 4a | 0 | 0 | 1 | 0.274 |
| **Cl2** | 4d | 0.25 | 0.25 | 0.75 | 0.626 |
| **P1** | 4b | 0 | 0 | 0.5 | 1 |
| **S1** | 4d | 0.25 | 0.25 | 0.75 | 0.374 |
| **S2** | 16e | 0.1186 | -0.1186 | 0.6186 | 1 |
| **S3** | 4a | 0 | 0 | 1 | 0.626 |
| **I1** | 4d | 0.25 | 0.25 | 0.75 | 0.1 |

**Table S4**. Summary of galvanostatic cycling conditions on lithium plating and stripping in sulfide-based symmetric cells.

| Electrolyte | Critical  Current  Density  (mA cm^-2^) | Cycling  Time (h) | Ref |
| --- | --- | --- | --- |
| LPSC-0.1LiI | 1.6 | 7000 | This work |
| Li_3.12_P_0.94_B_i0.06_S_3.91_O_0.09_ | 1.2 | 2000 | 1 |
| Li_5.5_P_0.96_Sb_0.04_S_4.4_O_0.1_Cl_1.5_ | 1.5 | 700 | 2 |
| LPSC-MF | 1.4 | 1800 | 3 |
| LPSCl-0.02Y | 0.8 | 1000 | 4 |
| LPSBr_1.5_-4%ZnO | 1.4 | 800 | 5 |
| Li_3.04_P_0.96_Zn_0.04_S_3.92_F_0.08_. | 1 | 550 | 6 |
| Li-LAF/ Li_6_PS_5_Cl /LAF-Li | 1.6 | 3000 | 7 |
| Li_6_P_0.925_Sb_0.075_S_5_Cl | 1.2 | 800 | 8 |

**References**

1. Y. Ni, C. Huang, H. Liu, Y. Liang, L. Fan, Adv. Funct. Mater. 2022, 32, 2205998.
2. C. Wei, C. Yu, R. Wang, L. Peng, S. Chen, X. Miao, S. Cheng, J. Xie, J. Power Sources 2023, 559, 232659.
3. C. Liu, B. Chen, T. Zhang, J. Zhang, R. Wang, J. Zheng, Q. Mao, X. Liu, Angew. Chem. Int. Ed. 2023, 62, 202302655.
4. Y. Xia, J. Li, J. Zhang, X. Zhou, H. Huang, X. He, Y. Gan, Z. Xiao, W. Zhang, J. Power Sources 2022, 543, 231846.
5. T. Chen, L. Zhang, Z. Zhang, P. Li, H. Wang, C. Yu, X. Yan, L. Wang, B, Xu, ACS Appl. Mater. Interfaces 2019, 11(43), 40808-40816.
6. Y. Gao, J. Gao, Z. Zhang, Y. Wu, X. Sun, F. Zhao, Y. Zhang, D. Song, W. Si, Q. Zhao, X. Yuan, J. Wu, ACS Appl. Mater. Interfaces 2024,16, 18843.
7. Q. Fan, W. Zhang, Y. Jin, D. Zhang, X. Meng, W. Peng, J. Wang, J. Mo, K. Liu, L. Liu, M. Li, Chem. Eng. J. 2023, 477, 147179.
8. H. Liu, Q. Zhu, Y. Liang, C. Wang, D. Li, X. Zhao, L. Gao, L. Fan, Chem. Eng. J. 2023, 462, 142183.

1. * Corresponding authors: [yaruliang@xtu.edu.cn](mailto:yaruliang@xtu.edu.cn)(Y.R.Liang);[gbxu@xtu.edu.cn](mailto:%20gbxu@xtu.edu.cn(G.B.Xu))(G.B.Xu); [↑](#footnote-ref-1)
2. [xingqiaowu@wzu.edu.cn (X.Q.Wu)](mailto:gbxu@xtu.edu.cn(X.Q.Wu)) [↑](#footnote-ref-2)
3. [↑](#footnote-ref-3)
